# Liver Enhancement on Computed Tomography Is Suboptimal in Patients with Liver Steatosis

**DOI:** 10.3390/jpm11121255

**Published:** 2021-11-25

**Authors:** Véronique V. van Cooten, Daan J. de Jong, Frank J. Wessels, Pim A. de Jong, Madeleine Kok

**Affiliations:** Department of Radiology, University Medical Center Utrecht, Heidelberglaan 100, 3584 CX Utrecht, The Netherlands; v.v.vancooten@students.uu.nl (V.V.v.C.); d.j.dejong4@students.uu.nl (D.J.d.J.); f.j.wessels-3@umcutrecht.nl (F.J.W.); P.deJong-8@umcutrecht.nl (P.A.d.J.)

**Keywords:** contrast media, liver, fatty liver, tomography, X-ray computed

## Abstract

This study’s aim was twofold. Firstly, to assess liver enhancement quantitatively and qualitatively in steatotic livers compared to non-steatotic livers on portal venous computed tomography (CT). Secondly, to determine the injection volume of contrast medium in patients with severe hepatic steatosis to improve the image quality of the portal venous phase. We retrospectively included patients with non-steatotic (*n* = 70), the control group, and steatotic livers (*n* = 35) who underwent multiphase computed tomography between March 2016 and September 2020. Liver enhancement was determined by the difference in attenuation in Hounsfield units (HU) between the pre-contrast and the portal venous phase, using region of interests during in three different segments. Liver steatosis was determined by a mean attenuation of ≤40 HU on unenhanced CT. Adequate enhancement was objectively defined as ≥50 ΔHU and subjectively using a three-point Likert scale. Enhancement of non-steatotic and steatotic livers were compared and associations between enhancement and patient- and scan characteristics were analysed. Enhancement was significantly higher among the control group (mean 51.9 ± standard deviation 11.5 HU) compared to the steatosis group (40.6 ± 8.4 HU *p* for difference < 0.001). Qualitative analysis indicated less adequate enhancement in the steatosis group: 65.7% of the control group was rated as good vs. 8.6% of the steatosis group. We observed a significant correlation between enhancement, and presence/absence of steatosis and grams of iodine per total body weight (TBW) (*p* < 0.001; adjusted R^2^ = 0.303). Deduced from this correlation, theoretical contrast dosing in grams of Iodine (g I) can be calculated: g I = 0.502 × TBW for non-steatotic livers and g I = 0.658 × TBW for steatotic livers. Objective and subjective enhancement during CT portal phase were significantly lower in steatotic livers compared to non-steatotic livers, which may have consequences for detectability and contrast dosing.

## 1. Introduction

Computed tomography is often used for the identification of focal liver lesions. A distinction can be drawn between hypervascular and hypovascular lesions in the liver. For detection of hypervascular lesions the arterial phase is crucial and similarly essential is portal phase imaging for detecting hypovascular lesions [1,2].

Detection of focal liver lesions can prove more difficult in steatotic livers. On unenhanced CT hypodense lesions, which are already difficult to detect in non-steatotic livers, may present as isodense in steatotic livers and may thus be missed [3]. This increases the importance of a sufficient contrast-enhanced CT in the presence of liver steatosis.

Steatotic liver disease is an umbrella term for non-alcoholic fatty liver disease and alcohol-related fatty liver disease, of which non-alcoholic fatty liver disease alone has a global prevalence of 25% [4]. The fact that most liver metastases are hypovascular and the high prevalence of non-alcoholic fatty liver disease emphasises the importance of protocol optimisation for the identification of hypovascular liver lesions on CT [5].

Generally, enhancement (ΔHU between unenhanced and enhanced CT) of liver parenchyma of ≥50 HU is considered adequate for detecting hypovascular liver lesions [6]. CT attenuation is influenced by numerous parameters, e.g., scan parameters, patient-related factors, and injection parameters [7]. In patients with chronic liver disease, there is a decrease in enhancement of the liver parenchyma during the portal venous phase which might cause the required enhancement of ≥50 HU not to be reached. Therefore, hypovascular lesions are possibly not adequately detected [6,8]. However, these studies often excluded patients with severe liver steatosis (≤40 HU). Additional research on enhancement of the liver parenchyma including severe steatotic livers might substantially contribute to improving further detection of hypovascular liver lesions [4].

This study’s aim was twofold. Firstly, to assess liver enhancement quantitatively and qualitatively in steatotic livers compared to non-steatotic livers on portal venous computed tomography. Secondly, to determine the injection volume of contrast medium in patients with severe hepatic steatosis to improve the image quality of the portal venous phase.

## 2. Materials and Methods

### 2.1. Patient Selection

The WMO, the Dutch Law on Medical Research, was not applicable to this study as reviewed by the local medical ethical committee (METC, ref. 20–025/C). Informed consent was waived for this retrospective review of patient records and images, due to the anonymous research data handling. 

Between March 2016 and September 2020, 884 patients underwent a multiphase abdominal CT with suspicion of a kidney tumour in the University Medical Center Utrecht. These scans were retrospectively reviewed. 

Inclusion criteria were: multi-phase contrast-enhanced CT (including unenhanced and portal venous phase images) of the abdomen, an age of ≥18 years and severe liver steatosis (unenhanced liver attenuation of ≤40 HU) [9,10]. General exclusion criteria were: technical difficulties during scanning, major artifacts (e.g., due to stents or implants, surgery), cirrhosis, numerous metastases, extensive liver cystic lesions, extensive peribiliary fibrosis and partial hepatectomy. 311 scans did not include an unenhanced phase and of these 573 remaining scans only 35 met the criterium of severe steatosis. None of these 35 scans met any of the exclusion criteria.

As a control group, 70 patients without liver steatosis were randomly selected from the same patient population from a previously performed study by De Jong et al. [11].

### 2.2. CT Scanning Protocols

The CT scans were performed on a Brilliance iCT 256, an IQon-spectral and a Brilliance 64 scanner (Philips Healthcare, Best, The Netherlands). CT examination was performed with a tube voltage of 120 kV and a reference tube current of 127 mAs, 116 mAs, and 102 mAs for the three scanners respectively. A gantry rotation time of 0.4 s, 0.27 s and 0.4 s respectively and a detector collimation of: 128 × 0.625 mm, 64 × 0.625 mm, and 64 × 0.625 mm respectively. For the unenhanced phases, images were displayed in the axial plane as 5 mm-thick sections with 4 mm increments. The portal venous phase images were displayed in axial, coronal, and sagittal plane, with 5 mm-thick slices and 4 mm increments. A B (abdominal) kernel at iDose level 3 was used to reconstruct the images. The scan range was the upper abdomen for the unenhanced phase. For the portal venous phase the range was set 1 cm superior of the diaphragm to the lower pelvis. 

Bolus tracking was used to determine imaging timing, by measuring a circular region of interest (ROI) in the abdominal aorta. Delay post-threshold (150 HU) before scanning was set at 90 s for the portal venous phase. 

### 2.3. Contrast Material Injection

All patients received preheated iodinated contrast (Ultravist, Iopromide 300 mg I/mL; Bayer Healthcare, Berlin, Germany) through an 18–20 Gauge cannula in an antecubital vein by use of a standard dual-head CT power injector (Stellant, Bayer Healthcare, Berlin, Germany). 

The following contrast protocol, based on weight categories, was used: ≤70 kg, 70–90 kg, and ≥90 kg. For the ≤70 kg group an injection volume of 120 mL with 36.0 g of Iodine (g I) at a flow rate of 4 mL/s was administered. For the 70–90 kg weight group 150 mL with 45.0 g I at 4.5 mL/s. For the ≥90 kg group 185 mL, 55.5 g I at a rate of 5 mL/s. Afterwards, a fixed 50 mL saline flush was injected at the same flow rate. 

### 2.4. Quantitative Image Analysis

During unenhanced and portal venous phase CT attenuation values (HU) were measured using circular regions of interest (ROIs). ROIs were located in segments 2, 7, and 8, based on Couinaud segmental classification, avoiding visible vessels, bile ducts and potential liver lesions. The mean attenuation values per phase were calculated. The change in attenuation, determined as enhancement (ΔHU), was calculated by subtracting pre-contrast values from the values measured during the portal venous phase. 

Based on enhancement values scans were divided into three groups: adequate (ΔHU ≥ 50 HU), moderate (ΔHU 40–50 HU) and insufficient (ΔHU ≤ 40 HU), as proposed by Koiwahara et al. [8].

### 2.5. Qualitative Image Analysis 

The subjective quality of all scans was independently assessed both by M.K. (5 years of experience in abdominal radiology) and by F.W. (12 years of experience in abdominal radiology). Scoring was done for liver enhancement. A three-point Likert scale was used for expressing the diagnostic value of liver enhancement: 1 = good; 2 = moderate (diagnostic); 3 = poor (non-diagnostic).

### 2.6. Statistical Analysis

All statistical tests were performed using the software SPSS version 26 (SPSS Inc., Chicago, IL, USA). All *p*-values are 2-sided and a *p*-value lower than 0.05 was considered to be statistically significant. 

Histograms and the Shapiro–Wilk test were used to assess distribution of data. For normally distributed variables, expressed as the mean with standard deviation (± SD), independent samples *t*-tests were used to compare groups. Mann–Whitney U tests were used for the non-parametric variables, expressed as the median with interquartile range (IQR). For categorical variables, reported as proportions, chi-square tests were used to compare groups. 

Single- and multivariable linear regression analyses were performed to predict ΔHU. Enhancement was analysed for patient characteristics and injection parameters, and additionally for g I/TBW, a method proposed by Heiken et al. [12]. Inclusion of variables in a multivariable linear regression model was based on the *p*-values of the single linear regression analysis of the variables, only including variables with a *p* < 0.05. Thereafter, excluding variables one-by-one starting with the highest individual *p*-value, until all variables in the multivariate model became significant (*p* < 0.05).

Cohen’s kappa (K) was used to determine inter-rater reliability for the qualitative image analysis.

## 3. Results

### 3.1. Baseline Characteristics

Shown in Table 1 is a summary of baseline characteristics. The patient characteristic sex did not differ significantly between the control group and liver steatosis group, 75.7% vs. 71.4% male. Patients in the control group had a higher median age (65.5 years) than the liver steatosis group (64 years); however, this difference was not statistically significant. In addition, the difference in median flow rate between the control group (4.5 mL/s) and the liver steatosis group (4.5 mL/s) was not significant. Moreover, the median iodine delivery rate (IDR), calculated on the basis of flow rate, was not significantly different between groups, 1.35 g I/s for the control group and 1.35 g I/s for the liver steatosis group. There was no statistically significant difference for mean length between the control group (1.77 m) and the liver steatosis group (1.72 m). No statistically significant difference was indicated for grams of Iodine (g I), which were both 45.0 g I. Lastly, there was no significant difference for injection duration, both groups had equal medians (33.3 s).

The median weight of the patients with liver steatosis was higher (92.0 kg) than the control group (81.0 kg), which differed significantly (*p* < 0.001). Similarly significant was the difference in median BMI between the control (26.0) and liver steatosis group (29.5), *p* < 0.001. 

The mean grams of Iodine per TBW was higher in the control group (0.53 g I/kg) than in the liver steatosis group (0.48 g I/kg), which was statistically significant, *p* = 0.002.

### 3.2. Quantitative Image Quality

Attenuation values during unenhanced and portal venous phase for segments 2, 7 and 8, and portal venous enhancement (ΔHU) are summarised in Table 2. For the control group mean attenuation during the unenhanced phase was higher (57.1 HU) than the median of the liver steatosis group (29.8 HU). The mean portal attenuation was similarly higher for the control group (108.9 HU) than for the liver steatosis group (70.4 HU). The difference in attenuation during these phases were all statistically significant (*p* < 0.001).

**Figure 1:**
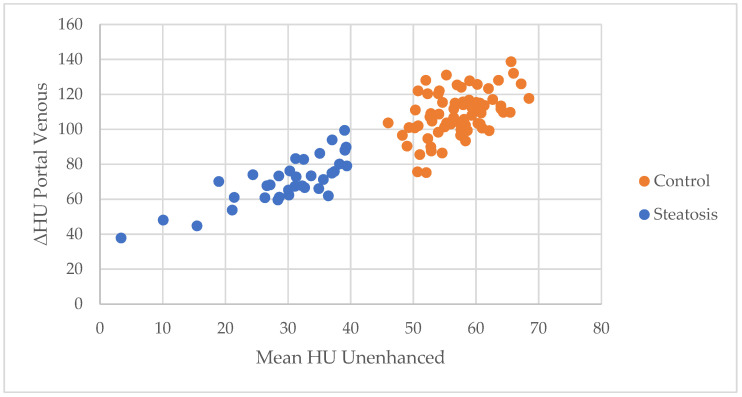
Scatter plot of enhancement (ΔHU) during the portal venous phase by mean unenhanced attenuation (HU) for control and liver steatosis group.

Enhancement (ΔHU) during the portal venous phase was significantly higher in the control group (51.9 HU) than in the liver steatosis group (40.6 HU), *p* < 0.001, also visualised in Figure 1. 

Adequacy of enhancement during the portal venous phase was evaluated by categorising scans into three groups based on HU-values. In Table 3 can be seen that in the control group 55.7% of the scans were labelled as adequate, whereas 20.0% of the scans in the liver steatosis group got this label. Furthermore, in the control group 30.0% of the scans were graded as moderate and 14.3% as insufficient, whilst these numbers were 22.9% and 57.1% in the liver steatosis group.

### 3.3. Qualitative Analysis 

Adequacy of enhancement according to the three-point Likert scale is stated in Table 4. For the control group 65.7% was rated as good, as moderate in 34.3%, as poor in 0.0%. For the steatosis group only 8.6% of the scans were graded as good, most (88.5%) were graded as moderate and 2.9% were graded as poor. 

The majority of the scans with a enhancement of ≥50 HU were subjectively graded as good (69.6%), 30.4% was graded as moderate and none as poor. Of the scans with an enhancement between 40–50 HU 44.8% was graded as good, 55.2% as moderate and none as poor.

The scans with a ΔHU ≤40 were graded as good in only 13.3%: the majority were graded as moderate in 83.3% and 3.3% as poor. 

Interobserver reliability of the qualitative image analysis was good for liver enhancement, with a kappa-values of 0.713. 

### 3.4. Regression Analysis

Simple linear regression was run to predict enhancement during the portal phase from baseline characteristics, see Table 5. For the association between ΔHU and grams of iodine per TBW (R^2^ = 0.213; *p* < 0.001), presence/absence of liver steatosis (R^2^ = 0.205; *p* < 0.001), weight (R^2^ = 0.122; *p* < 0.001), and BMI (R^2^ = 0.057; *p* = 0.017), a statistically significant correlation was observed. The parameters age (*p* = 0.996), length (*p* = 0.087), grams of Iodine (*p* = 0.357), flow rate (*p* = 0.978), IDR (*p* = 0.978), and injection duration (*p* = 0.257) had no significant correlation with ΔHU. 

The formula for the relationship between enhancement and grams of iodine per TBW, a method proposed by De Jong et al., was: ΔHU = 10.331 + 73.340 × g I/TBW which can also be written as g I = TBW × (ΔHU − 10.331)/73.340 [11].

A multiple regression model including grams of iodine per TBW and presence/absence of liver steatosis statistically significantly predicted ΔHU, F(2, 96) = 22.328; *p* < 0.001; adjusted R^2^ = 0.303. Models including the parameters weight, length, and/or BMI did not add statistically significantly. 

### 3.5. Predicted Dose of Contrast Media

The formula corresponding with the above discussed significant model is ΔHU = 21.638 + 56.549 × g I/TBW − 8.824 × Steatosis, in which steatosis can be defined as 0 = no, in the absence of liver steatosis and 1 = yes, when it is present. 

Based on the earlier discussed adequate enhancement of ≥50 HU needed for detection of hypovascular liver lesions, the formula can be transformed to:

g I = TBW × (28.362 + 8.824 × Steatosis)/(56.549) ≈ (0.502 + 0.156 × Steatosis) × TBW

Formulas for grams of Iodine are g I = 0.502 × TBW for non-steatotic livers and g I = 0.658 × TBW for steatotic livers. Based on these equations steatotic livers need Δg I = 0.156 per kg TBW more compared to non-steatotic livers, corresponding to an additional 0.52 cc of contrast medium per kg TBW.

## 4. Discussion

In this study, we observed worse enhancement during the portal venous phase, both objectively and subjectively in the presence of liver steatosis when compared to non-steatotic livers. In order to establish sufficient enhancement, according to previous literature ≥50 HU, patients with liver steatosis might need a higher amount of iodine per kilogram of body weight compared to patients without steatosis. In subsequent studies the effect on lesion detection remains to be characterised.

Inadequately enhanced (<50 HU) livers were found more often in the liver steatosis group, compared to the livers of patients without liver steatosis. This also corresponded with subjective assessments. To our knowledge, no similar studies have been conducted on the characterisation of liver enhancement in steatotic livers. There is a study with a similar design by Koiwahara et al., although they investigated the influence of liver cirrhosis on enhancement [8]. They also showed a decrease in the adequacy of portal venous enhancement cirrhotic livers with Child-Pugh grades A and B, when compared to non-cirrhotic livers. These results can partly be compared to our results as changes in blood flow in steatotic livers are comparable to those of cirrhotic livers [13]. The portal phase is mostly determined by the portal perfusion. In patients with cirrhotic livers, portal hypertension due to fibrotic changes cause a reduction in portal perfusion [14]. In liver steatosis, portal hypertension can also occur, even when there a no signs of fibrosis [13]. This rise in portal pressure is speculated to be caused by a reduction in sinusoidal flow, hence increasing intrahepatic resistance [13,15]. The flow reduction has been demonstrated to be caused by liver parenchymal cells swollen with lipids, which narrow the sinusoidal lumen [15]. These changes in portal perfusion could be the reason of less adequate enhancement in steatotic livers. In steatohepatitis there are fibrotic changes which could additionally contribute to portal hypertension and subsequent less adequate enhancement [16]. 

Furthermore, in the study of Koiwahara et al. they also observed a decrease in enhancement as the severity of cirrhosis increased. 

In our study with limited patients, we also observed a visual relationship between the amount of fatty infiltration and the enhancement of the liver. The majority (57.1%) of the patients with steatosis had an unenhanced attenuation between 30–40 HU. The mean of attenuation for this group was 29.8 (SD ± 8.4). A relationship between severity of steatosis and decrease in enhancement can be carefully speculated upon by examining Figure 1. Nonetheless, as a result of this limited range, no definitive statements can be made. More severe degrees of steatosis are more associated with portal hypertension [13]. Thus, the decrease in enhancement for steatotic livers could have similar pathogenesis to cirrhotic livers. 

Apart from presumed abnormal blood flow in steatotic livers causing lower enhancement, we found some significant differences in patient and contrast media injection related parameters that might have influenced the differences in enhancement between both groups. A significantly lower amount of iodine per total body weight (g I/TBW) was found for the liver steatosis group. As all patients received the amount of contrast media according to a semi-fixed injection protocol divided by three weight groups, it is to be expected that this significant difference is caused by the significant difference in total body weight between liver steatosis group and the control group, as a higher body weight was found for patients with liver steatosis. This amplifies the results from previous studies about per-patient body weight-adapted contrast injection protocols showing more homogenous enhancement between patients in comparison to (semi) fixed contrast volume protocols [11,17]. Furthermore, enhancement of livers could be evaluated for other contrast dosing protocols, e.g., lean body weight (LBW), BMI, or body surface area [8,11]. A protocol on the basis of lean body weight has been suggested, but has proved similar to a protocol based on TBW [18]. Furthermore, de Jong et al. has shown little additional value of LBW for contrast dosing protocols based on TBW [11,19].

The finding that steatotic livers enhance poorly is of importance, because patients with liver steatosis are potentially at risk for the underdetection of hypovascular liver lesions as found by Nakai et al. [20]. This was attributed to hypodense metastases presenting as isodense lesions in a relatively low attenuating fat infiltrated background [3,20]. Moreover, as was found in our study, the decreased enhancement of steatotic livers might also contribute to inadequate detection of lesions. To prevent steatotic livers from enhancing inadequately, we theorise patients with liver steatosis need an extra amount of contrast media per kilogram of TBW to establish sufficient enhancement (≥50 HU).

We also performed a subjective analysis wherein we graded the perceived enhancement of steatotic livers compared to non-steatotic livers. We found that moderately enhancing scans (40–50 HU) were still graded as good in 44.8%, as moderate in 55.2% and none as poor. The scans labelled as enhancing insufficiently (≤40 HU) were subjectively graded as good in 13.3%, the majority, 83.3%, were graded as moderate, and 3.3% as poor. Even though an enhancement of ≥50 HU is a commonly used threshold of adequacy of enhancement, an enhancement below 50 HU might still be of diagnostic value [6,8]. This corresponds with the findings of de Jong et al. in which is stated that an enhancement of ≥40 HU could be considered to be of diagnostic value [11]. Thus, even for patients with previously diagnosed steatotic liver disease, increased contrast dosing might not be necessary for current CT scans [21]. The contrast dose as suggested by the regression analysis might be an overestimation and should possibly be limited to non-diagnostic scans only, for example by lowering the threshold to 40 HU instead of 50 HU. However, this needs to be further investigated in a clinical study taking lesion detection into account.

Furthermore, it is also possible that due to before discussed sinusoidal and flow changes, steatotic livers are unable to enhance sufficiently, even when increasing the contrast dose. Therefore, alternatives for improving enhancement in CT other than increasing the iodine dose should be further explored, e.g., lower voltage without increasing image noise or virtual monochromatic imaging with low kV reconstruction [7,22]. Alternatively, switching from CT to MRI could be considered for patients with liver steatosis, because of an increased sensitivity of detection and classification of liver lesions in MRI compared to CT [23,24,25].

There are some limitations to our study. Firstly, the TBW did significantly differ between the control group and the steatosis group. The TBW is an influential factor in the enhancement of patients [7]. As expected, the steatotic liver group was heavier, because the prevalence and the degree of steatosis corresponds with the grade of obesity [26]. This difference might have been of importance to our study, but based on multivariate linear regression, the steatosis group still required more grams of Iodine per kg total body weight. This means that steatosis independently contributes to a decrease in enhancement. Secondly, only a limited amount of patients (*n* = 35) with liver steatosis who underwent multiphase computed tomography could be identified from 2016 to 2020. More recent patients could not be included due to revision of the contrast dosing protocol. In addition, we do not have additional patient characteristics given the retrospective nature of the study and privacy regulations. Moreover, a limitation of this study was that CT scanning factors were not uniform since scans were retrospectively reviewed from four different scanners, which could have caused interscanner variability [27]. However, the most important factor influencing attenuation values is the tube voltage (kV), which was set to 120 kV for each scanner, making influence on attenuation values negligible in this study, which limits interscanner variability.

Another downside of this study was that the diagnosis of liver steatosis in this research was based on imaging alone and not biopsy and histological evaluation, often considered the reference standard for the assessment of non-alcoholic fatty liver disease [28,29]. However, the method of using the absolute attenuation ≤40 HU of the liver to diagnose sever liver steatosis has a high specificity and thus serves as an reliable alternative for the diagnosis [9]. Differentiation between different types of liver steatosis was not within the scope of this study. Neither differentiation between non-alcoholic and alcoholic causes of liver steatosis nor differentiation between simple steatosis and steatohepatitis were made, which could confound results [21]. 

## 5. Conclusions

In conclusion, enhancement during the portal venous phase was significantly lower in patients with steatotic livers compared to non-steatotic livers and subjective image quality decreased in the presence of liver steatosis. Regression analysis showed a higher indicated iodine dose per total body weight for steatotic livers compared to non-steatotic livers. Future research should focus on possible adaptations in contrast media administration or alternatives to CT for improved enhancement in livers with liver steatosis. 

## Figures and Tables

**Table 1:** Baseline characteristics.

Characteristic	Control	Liver Steatosis	*p*-Value
No. participants	70	35	-
Sex male	53 (75.7%)	25 (71.4%)	0.636 ^a^
Age (years)	65.5 (55.8–73.0)	64.0 (57.0–69.0)	0.273 ^b^
Length (m)	1.77 (1.70–1.83)	1.72 (1.68–1.83)	0.360 ^b^
Weight (kg)	81.0 (73.0–88.0)	92.0 (84.0–103.0)	<0.001 ^b^
BMI	26.0 (23.8–28.9)	29.5 (26.2–35.7)	<0.001 ^b^
Grams of iodine (g I)	45.0 (39.0–45.0)	45.0 (43.5–45.0)	0.080 ^b^
Grams of iodine/TBW (g I/kg)	0.53 (±0.074)	0.48 (±0.066)	0.002 ^c^
Flow rate (ml/s)	4.5 (4.0–4.5)	4.5 (4.5–4.75)	0.187 ^b^
Iodine delivery rate (g I/s)	1.35 (1.20–1.35)	1.35 (1.35–1.43)	0.187 ^b^
Injection duration (s)	33.3 (33.3–33.3)	33.3 (31.2–33.3)	0.154 ^b^

Mean (± SD) or median (IQR). ^a^ Group difference was tested using a chi-square test. ^b^ Group difference was tested with the Mann–Whitney U test. ^c^ Group difference was testing with the independent samples *t*-test.

**Table 2:** Comparison of attenuation during unenhanced and portal phase in segments 2, 7, and 8.

Phase	Segment	Control (*n* = 70)	Liver Steatosis (*n* = 35)	*p*-Value
Unenhanced	S2	58.6 (± 4.9)	32.2 (± 9.3)	
	S7	55.8 (± 5.5)	29.1 (± 8.2)	
	S8	56.9 (± 5.6)	28.2 (± 9.9)	
	Mean	57.1 (± 5.0)	29.8 (± 8.4)	<0.001
Portal	S2	109.8 (± 13.2)	73.5 (± 12.8)	
	S7	107.2 (± 12.9)	69.2 (± 13.4)	
	S8	109.8 (± 14.4)	68.6 (± 15.7)	
	Mean	108.9 (± 13.0)	70.4 (± 13.3)	<0.001
ΔHU portal venous ^a^		51.9 (± 11.5)	40.6 (± 8.4)	<0.001

Data presented as mean (±SD) or median (IQR). *p*-values were calculates using Mann–Whitney tests. ^a^ ΔHU was calculated by subtracting the unenhanced mean value of S2, S7, and S8 from the enhanced portal venous, mean value of S2, S7, and S8.

**Table 3:** Adequacy of liver enhancement during the portal phase based on enhancement values.

Enhancement	Label	Control (*n* = 70)	Steatosis (*n* = 35)
≥50 HU	Adequate	39 (55.7%)	7 (20.0%)
40–50 HU	Moderate	21 (30.0%)	8 (22.9%)
≤40 HU	Insufficient	10 (14.3%)	20 (57.1%)

Data presented as the number (percentage) of patients.

**Table 4:** Subjective adequacy analysis of enhancement.

Label	Control (*n* = 70)	Steatosis (*n* = 35)
Good	46 (65.7%)	1 (8.6%)
Moderate	24 (34.3%)	33 (88.5%)
Poor	0 (0.0%)	1 (2.9%)

Data presented as the number (percentage) of patients.

**Table 5:** R^2^ in single and multiple linear regression.

Variable	Simple R2 |^a^	*p*-Value	Multiple Adjusted R2 |^b^	Significant F Change
Grams of Iodine per TBW	0.213	<0.001	0.205	<0.001
Liver steatosis	0.205	<0.001	0.303	<0.001
Weight	0.122	<0.001	0.310	0.174
BMI	0.057	0.017		
Length	0.029	0.087		
Injection duration	0.013	0.257		
Grams of Iodine	0.008	0.357		
Flow rate/IDR	<0.001	0.978		
Age	<0.001	0.996		

^a^ An individual R2 in simple linear regression. ^b^ Adjusted R2 when added to a multiple linear regression model together with the variables above.

## Data Availability

The data presented in this study are available on request from the corresponding author. The data are not publicly available due to ongoing unpublished research.
